# Factors Associated With Long Survival in Patients With Cancer Admitted to Palliative Care: An Exploratory Study

**DOI:** 10.1177/08258597241231005

**Published:** 2024-02-19

**Authors:** José António Ferraz-Gonçalves, Adelaide Alves, Álvaro José Silva, Ana Carmo Valente, Ana Pina, Áurea Lima, Daniela Antunes, Francisco Cubal, Isabel Costa, Jorge Rodrigues, Mariana Costa, Mariana Ramos, Michael Luis, Sofia Garcês Soares, Sofia Sousa, Teresa Dias Moreira, Vânia Sá-Araújo, Maria José Bento

**Affiliations:** 1 Faculty of Medicine, Porto University, Porto, Portugal; 2 Department of Palliative Care, Portuguese Oncology Institute of Porto, Porto, Portugal; 3 Department of Pneumology, Centro Hospitalar de Vila Nova de Gaia/Espinho, Vila Nova de Gaia, Portugal; 4 Condestável Family Health Unit, Department of General and Family Medicine, Batalha, Portugal; 5 Department of Medical Oncology, Centro Hospitalar e Universitário de S. João, Porto, Portugal; 6 Department of Medical Oncology, Portuguese Oncology Institute of Lisbon, Lisbon, Portugal; 7 Department of Medical Oncology, Portuguese Oncology Institute of Porto, Porto, Portugal; 8 CESPU, Cooperativa de Ensino Superior Politécnico e Universitário, Gandra, Portugal; 9 Centro Hospitalar de Leiria, Leiria, Portugal; 10 Department of Clinical Hematology, Centro Hospitalar de Trás-os-Montes e Alto Douro, Vila Real, Portugal; 11 Department of Medical Oncology, Braga Hospital, Braga, Portugal; 12 Department of Internal Medicina, Hospital de Santarém, Santarém, Portugal; 13 Department of Internal Medicine, Centro Hospitalar Tâmega e Sousa, Penafiel, Portugal; 14 Serviço de Pneumologia do Hospital de Braga, Braga, Portugal; 15 Group of Epidemiology, Results, Economy and Management in Oncology, IPO Porto Research Center (CI-IPOP), Portuguese Oncology Institute of Porto (IPO Porto), Portugal; 16 Department of Epidemiology, Portuguese Oncology Institute of Porto (IPO Porto), Porto, Portugal.; 17 Population Studies Department. School of Medicine and Biomedical Sciences, ICBAS, University of Porto, Porto, Portugal

**Keywords:** prognosis, palliative care, long survival, patients with cancer

## Abstract

**Objective:** Some patients with cancer admitted to palliative care have relatively long survivals of 1 year or more. The objective of this study was to find out factors associated with prolonged survival. **Methods:** Retrospective case-control study comparing the available data of patients with cancer who survived more than 1 year after admission in a palliative care service with patients with cancer who survived 6 months or less. The intended proportion was 4 controls for each case. Patients were identified through electronic records from 2012 until 2018. **Results:** And 1721 patients were identified. Of those patients, 111 (6.4%) survived for at least 1 year, and 363 (21.1%) were included as controls according to the established criteria. The intended proportion could not be reached; the proportion was only 3.3:1. The median survival of cases was 581 days (range: 371-2763), and the median survival of controls was 57 days (range: 1-182). In the multivariable analysis, patients with a hemoglobin ≥ 10.6 g/dL and a creatinine level >95 µmol/L had a higher probability of living more than 1 year. In contrast, patients with abnormal cognition, pain, anorexia, liver metastases, an Eastern Cooperative Oncology Group performance status >1, and a neutrophil/lymphocyte ratio ≥ 3.43 had a low probability of living more than 1 year. **Conclusion:** Several factors were statistically associated positively or negatively with prolonged survival. However, the data of this study should be confirmed in other studies.

## Introduction

The amount of survival is not the primary aim of palliative care. Nevertheless, calculating the survival of patients is essential for several reasons. It is crucial for patients and their families to plan their lives and allow them to solve issues such as settling financial matters and saying their goodbyes to their loved ones. It is also essential for health professionals to judge the adequacy of interventions, such as proceeding with transfusions, initiating antibiotics, or concentrating on comfort measures. In some countries, survival is essential for reimbursement for medical services.^1^

However, prognostication is uncertain, so survival might be longer or shorter than expected. The survival of patients referred to palliative care is usually expected to be relatively short. For example, the surprise question used frequently to identify patients for palliative care uses different time frames for the expected survival.^2^ However, 1 year is the longest used,^3^ to the best of our knowledge. The studies on prognosis performed in palliative care aim to determine how short survival seems.^4^ Some even try to find signs of impending death.^5^

Palliative care services (PCS) are often overwhelmed by admission requests, and there is a need to establish priorities, and the expected survival can be crucial in the decision process. In our PCS, many patients had relatively long survivals, sometimes several years, but patients with more than 1 year of follow up were not rare. For that reason, this study was carried out aiming to quantify them and to identify prognostic factors associated with those longer survivals, comparing the available factors recorded in patients’ files who lived more than 1 year with the same factors of patients who lived 6 months or less, a criterium that was once used to define a terminal patient.^6^ Therefore, the aim of this study is not about factors associated with short survival; on the contrary, this study aims to find prognostic factors of relatively long survival. To our knowledge, this is the first study that deals with this question.

## Methods

### Study Design

This is a retrospective case-control study. Patients were identified through electronic records from 2012 (the starting year of using electronic records) until December 2018. All patients were older than 18 years with solid tumors. Cases were all patients who survived longer than 1 year after being admitted to the PCS and had already died. As controls, patients had survival ≤6 months after admission on the PCS. Control patients were matched to cases by cancer type and sex, and in descending order:

- age (the limit was age more or less than 5 years from the case) and
- year of diagnosis (also more or less than 5 years).

When the criteria of age and year of diagnosis could not be respected, the patients closer to those criteria were selected. For patients with more than one cancer diagnosis, the last cancer diagnosed was considered the primary, except for nonmelanoma skin cancer, where the primary was the next-to-last.

### Setting

This study was carried out in a PCS of a public oncology hospital where around 10 000 new patients are admitted each year. The PCS was founded in 1994. Patients are referred to the PCS by physicians from the surgery, medical oncology, and radiotherapy services of the hospital. After being admitted to the PCS, patients usually are not submitted to antineoplastic treatment besides single fractions or short courses of radiotherapy for symptom control, such as metastatic bone pain or hemorrhage. Patients may be followed as inpatients, outpatients, or at home, depending on patients’ needs.

### Data Collection

Data studied were collected at admission to the PCS, except for serum hematological and biochemical parameters, which were the closest to the admission, as most patients did not undergo blood tests, at least in the first contact. Data collected included demographics, symptoms, other problems (such as ascites and pressure ulcers), Eastern Cooperative Oncology Group (ECOG) performance status, Charlson Comorbidity Index, social data, blood tests, date of diagnosis, date of the first observation in the PCS, initial context (out or inpatient), and date of death. Cognition was categorized as normal or abnormal based on the notes in patients’ files: if, in the record at admission, there was any reference to abnormal cognition, such as confusion, agitation, impaired consciousness, or another similar expression, cognition was classified as abnormal; otherwise, it was classified as normal.

### Statistical Analysis

For statistical purposes, initially, data were analyzed to identify coding errors, inconsistencies, and the presence of misclassification of cases or controls, and corrections were made where needed. An exploratory analysis of the data was performed to describe the sample. Variables were excluded if there were more than 15% missing. Continuous variables were categorized in quartiles. A logistic regression was used for univariable and multivariable analyses, and a confidence interval at 95% was calculated. The level of significance was deemed to be 0.05. In the univariable analysis, the Bonferroni correction was used to compensate for the number of variables. In the multivariable analysis, the Variance Inflation Factor was assessed to test for multicollinearity. The 29.0 version of the IBM SPSS statistical software was used to analyze the data.

## Results

In the period under consideration, a total of 1721 patients were identified through the administrative records of the hospital. Of those patients, 111 (6.4%) were followed for at least 1 year and were included as cases. As controls, 363 patients were identified according to the established criteria. The objective was to match 4 controls for 1 case, but only a proportion of 3.3:1 was reached. The median survival of cases was 581 days (range: 371-2763), and the median survival of controls was 57 days (range: 1-182). Table 1 presents demographic data. The intended proportion of primary diagnosis between cases and controls could not be reached in some cases; some of them do not have any control, and in thyroid cancer, the number of cases was higher than in controls.

**Table 1.** Demographic Data.

	Total, n (%)	Cases, n (%)	Controls, n (%)
First observation			
Outpatient	341 (72)	99 (89)	242 (67)
Inpatient	133 (28)	12 (11)	121 (33)
Gender			
Male	237 (50)	50 (45)	187 (52)
Female	237 (50)	61 (55)	176 (49)
Age quartiles (years)			
<65	109 (23)	19 (17)	90 (25)
65-75	125 (26)	24 (22)	101 (28)
76-81	115 (24)	24 (22)	91 (25)
>81	125 (26)	44 (40)	81 (22)
Primary cancer			
Colorectal	152 (32)	33 (30)	119 (33)
Stomach	49 (10)	10 (9)	39 (11)
Lung	40 (8)	8 (7)	32 (9)
Prostate	33 (7)	8 (7)	25 (7)
Head and neck	27 (6)	5 (5)	22 (6)
Cervix	24 (5)	6 (5)	18 (5)
Sarcoma	25 (5)	7 (6)	18 (5)
Breast	19 (4)	5 (5)	14 (4)
Kidney	16 (3)	3 (3)	13 (4)
Esophagus	14 (3)	3 (3)	11 (3)
Melanoma	14 (3)	4 (4)	10 (3)
Bladder	13 (3)	3 (3)	10 (3)
Body of the uterus	13 (3)	2 (2)	11 (3)
Liver	8 (2)	2 (2)	6 (2)
Brain	8 (2)	2 (2)	6 (2)
Thyroid	7 (2)	5 (5)	2 (1)
Female genital	4 (1)	1 (1)	3 (1)
Mesothelioma	3 (1)	1 (1)	2 (1)
Testicle	3 (1)	1 (1)	2 (1)
Fallopian tube	1 (0.2)	1 (1)	0 (0)
Anal canal	1 (0.2)	1 (1)	0 (0)
Cancer metastization			
Locally advanced	201 (43)	41 (37)	160 (44)
Lymph nodes	190 (40)	41 (37)	149 (41)
Liver	141 (30)	19 (17)	122 (34)
Lung	155 (33)	35 (32)	120 (33)
Peritoneal	72 (15)	11 (10)	61 (17)
Bone	100 (21)	20 (18)	80 (22)
Brain	34 (7)	3 (3)	31 (9)
Charlson Comorbidity Index			
0	166 (35)	32 (29)	134 (37)
1	115 (24)	25 (23)	90 (25)
2	90 (19)	22 (20)	68 (19)
≥3	102 (22)	32 (29)	70 (19)
ECOG performance status			
1	64 (14)	37 (33)	27 (8)
2	130 (29)	33 (30)	97 (29)
3	185 (41)	30 (27)	155 (46)
4	71 (16)	11 (10)	60 (18)

ECOG=Eastern Cooperative Oncology Group.

Table 2 shows the proportion of cases relative to the total number of patients identified in the study period. Some cancer types, such as pancreas, biliary ducts, and unknown primary, are not represented in cases.

**Table 2.** Percentage of Primary Cancers of Cases Relatively to the Total Number of Patients Admitted to Palliative Care.

Primary Cancer	Cases	Total	%
Fallopian tube	1	1	100
Thyroid	5	10	50
Mesothelioma (peritoneum)	1	3	33
Testicle	1	3	33
Sarcoma	7	47	15
Cervix	6	55	11
Colorectal	33	295	11
Melanoma	4	45	9
Brain	2	18	11
Anal canal	1	11	9
Kidney	3	44	7
Bladder	3	44	7
Liver	2	30	7
Prostate	8	132	6
Stomach	10	206	5
Lung	8	170	5
Female genital	1	22	5
Body of the uterus	2	46	4
Esophagus	3	78	4
Breast	5	113	4
Head and neck	5	151	3
Pancreas	0	44	0
Appendix	0	5	0
Small bowel	0	6	0
Primary unknown	0	16	0
Ureter	0	3	0
Ovarium	0	29	0
Biliary tract	0	40	0
Other	0	54	0
Total	111	1721	7

The results from the univariable analysis are presented in Tables 3A to 3D. The level of significance was calculated considering the number of variables, which was 24, using the Bonferroni correction. According to this correction, only variables with an adjusted *P* < .002 (0.05/24 = 0.002) were deemed significant. Therefore, only those variables were selected for the multivariable analysis. Of the general data, being observed for the first time as an inpatient, abnormal cognition, ECOG performance status higher than 1, and needing help for self-care were significantly associated with survival <6 months (Table 3A). Concerning the disease extension, liver metastases and the number of metastatic sites were significantly associated with controls (Table 3B). Several symptoms and problems were associated with a low probability of survival > 1 year, including pain, anorexia, nausea/vomiting, and the number of symptoms and other problems (Table 3C). Most available serum hematological and biochemical parameters, except potassium, lymphocytes, and platelets, were linked to survival (Table 3D).

**Table 3A.** Univariable Analysis by Logistic Regression—General Data.

Variable	Total, n (%)	Cases, n (%)	Controls, n (%)	OR	95% CI	*P*-value
First observation						
Outpatient	341 (72)	99 (89)	242 (67)	1		
Inpatient	133 (28)	12 (11)	121 (33)	**0**.**24**	**0.13-0.46**	**<**.**001**
Gender						
Male	237 (50)	50 (45)	187 (52)	1		
Female	237 (50)	61 (55)	176 (49)	1.30	0.85-1.99	.233
Age (years)						
<65	109 (23)	19 (17)	90 (25)	1		
65-75	125 (26)	24 (22)	101 (28)	1.13	0.58-2.19	.728
76-81	115 (24)	24 (22)	91 (25)	1.25	0.64-2.44	.514
>81	125 (26)	44 (40)	81 (22)	2.57	1.39-4.77	.003
Charlson Comorbidity Index						
0	166 (35)	32 (29)	134 (37)	1		
1	115 (24)	25 (23)	90 (25)	1.16	0.65-2.09	.614
2	90 (19)	22 (20)	68 (19)	1.36	0.73-2.51	.334
≥3	102 (22)	32 (29)	70 (19)	1.91	1.08-3.38	.025
Cognition						
Normal	345 (73)	95 (86)	250 (69)	1		
Abnormal	129 (27)	16 (14)	113 (31)	**0**.**37**	**0.21-0.66**	**<**.**001**
ECOG						
1	64 (14)	37 (33)	27 (8)	1		
2	130 (29)	33 (30)	97 (29)	**0**.**25**	**0.13-0.47**	**<**.**001**
3	185 (41)	30 (27)	155 (46)	**0**.**14**	**0.08-0.27**	**<**.**001**
4	71 (16)	11 (10)	60 (18)	**0**.**13**	**0.06-0.30**	**<**.**001**
Self-care						
Independent	95 (20)	42 (39)	53 (15)	**1**		
Some difficulty	55 (12)	16 (15)	39 (11)	0.52	0.26-1.05	.069
Needs a little Help	90 (19)	17 (16)	73 (20)	**0**.**29**	**0.15-0.57**	**<**.**001**
Needs a lot of Help	150(32)	25 (23)	125 (35)	**0**.**25**	**0.14-0.46**	**<**.**001**
Unable	80 (17)	9 (8)	71 (20)	**0**.**16**	**0.07-0.36**	**<**.**001**
Interval diagnosis-admission in palliative care (days)						
< 270	124 (27)	26 (24)	98 (27)	1		
270-708	110 (24)	17 (16)	93 (26)	0.69	0.35-1.35	.279
709-1478	119 (25)	24 (22)	95 (26)	0.95	0.51-1.177	.877
>1478	115 (25)	41 (38)	74 (21)	2.09	1.17-3.72	.012

*P*-value from logistic regression.

Bold—selected for multivariable analysis. The level of significance corrected by the Bonferroni method was <0.002.

CI=confidence interval; ECOG=Eastern Cooperative Oncology Group; OR=odds ratio.

**Table 3B.** Univariable Analysis by Logistic Regression—Disease Extension.

Variable	Total, n (%)	Cases, n (%)	Controls, n (%)	OR	95% CI	*P*-value
Locally advanced						
No	271 (57)	69 (63)	202 (56)	1		
Yes	201 (43)	41 (37)	160 (44)	0.75	0.48-1.16	.199
Lymph nodes						
No	282 (60)	69 (63)	213 (59)	1	
Yes	190 (40)	41 (37)	149 (41)	0.85	0.55-1.32	.467
Liver						
No	331 (70)	91 (83)	240 (66)	1		
Yes	141 (30)	19 (17)	122 (34)	**0.41**	**0.24-0.71**	.**001**
Lung						
No	317 (67)	75 (68)	242 (67)	1		
Yes	155 (33)	35 (32)	120 (33)	0.94	0.60-1.49	.795
Peritoneal						
No	400 (85)	99 (90)	301 (83)	1		
Yes	72 (15)	11 (10)	61 (17)	0.55	0.28-1.08	.084
Bone						
No	370 (79)	90 (82)	280 (78)	1		
Yes	100 (21)	20 (18)	80 (22)	0.78	0.45-1.34	.366
Brain						
No	438 (93)	107 (97)	331 (91)	1		
Yes	34 (7)	3 (3)	31 (9)	0.30	0.09-0.99	.050
Number of sites of metastasis						
0-1	199 (42)	60 (55)	139 (39)	1		
2	141 (30)	33 (30)	108 (30)	0.71	0.43-1.16	.170
≥3	130 (28)	17 (16)	113 (31)	**0.35**	**0.19-0.63**	**<**.**001**

*P*-value from logistic regression.

Bold—selected for multivariable analysis. The level of significance corrected by the Bonferroni method was <0.002.

CI=confidence interval; OR=odds ratio.

**Table 3C.** Univariable Analysis by Logistic Regression—Symptom/Problems.

Variable	Total, n (%)	Cases, n (%)	Controls, n (%)	OR	95% CI	*P*-value
Pain						
No	156 (33)	54 (49)	102 (28)	1		
Yes	318 (67)	57 (51)	261 (72)	**0**.**41**	**0.27-0.64**	**<**.**001**
Dyspnea						
No	391 (83)	89 (80)	302 (83)	1		
Yes	83 (18)	22 (20)	61 (17)	1.22	0.71-2.10	.465
Fatigue						
No	277 (58)	73 (66)	204 (56)	1		
Yes	197 (42)	39 (34)	159 (44)	0.67	0.43-1.04	.074
Anorexia						
No	299 (63)	94 (85)	205 (57)	1		
Yes	175 (37)	17 (15)	158 (44)	**0**.**24**	**0.13-0.41**	**<**.**001**
Nausea/vomiting						
Yes	75 (16)	9 (8)	66 (18)	1		
No	399 (84)	102 (92)	297 (82)	0.40	0.19-0.83	.013
Depression/sadness						
No	391 (83)	93 (84)	298 (82)	1		
Yes	83 (18)	18 (16)	65 (18)	0.89	0.50-1.57	.682
Anxiety						
No	420 (89)	97 (87)	323 (89)	1		
Yes	54 (11)	14 (13)	40 (11)	1.17	0.61-2.23	.644
Drowsiness						
No	442 (93)	109 (98)	333 (92)	1		
Yes	32 (7)	2 (2)	30 (8)	0.20	0.05-0.87	.031
Insomnia						
No	389 (82)	94 (85)	295 (81)	1		
Yes	85 (18)	17 (15)	68 (19)	0.79	0.44-1.40	.412
Constipation						
No	359 (76)	89 (80)	270 (75)	1		
Yes	114 (24)	22 (20)	92 (25)	0.73	0.43-1.23	.235
Number of symptoms						
0-1	104 (22)	40 (36)	64 (18)	1		
2	101 (21)	29 (26)	72 (20)	0.64	0.36-1.16	.141
3	108 (23)	18 (16)	90 (25)	**0**.**32**	**0.17-0.61**	**<**.**001**
≥4	161 (34)	24 (222)	137 (38)	**0**.**28**	**016-0.50**	**<**.**001**
Other problems						
0	285 (60)	78 (70)	207 (57)	1		
1	142 (30)	27 (24)	115 (32)	0.62	0.38-1.02	.060
2	47 (10)	6 (5)	41 (11)	0.39	0.16-0.95	.038

*P*-value from logistic regression.

Bold—selected for multivariable analysis. The level of significance corrected by the Bonferroni method was <0.002.

CI=confidence interval; OR=odds ratio.

**Table 3D.** Univariable Analysis by Logistic Regression—Serum Hematological and Biochemical Parameters.

Variable	Total, n (%)	Cases, n (%)	Controls, n (%)	OR	95% CI	*P*-value
Hemoglobin (g/dL)						
<9.1	98 (24)	7 (9)	91 (27)	1		
9.1-10.5	106 (26)	15 (19)	91 (27)	2.14	0.84-5.50	.113
10.6-11.9	100 (24)	23 (30)	76 (23)	4.11	1.68-10.05	.002
≥ 12.0	111 (27)	33 (42)	78 (23)	**5**.**50**	**2.31-13.13**	**<**.**001**
Leucocytes (×109/L)						
<6.76	100 (24)	31 (40)	69 (21)	1		
6.77-8.89	104 (25)	26 (34)	78 (23)	0.74	0.40-1.37	.34
8.90-12.32	103 (25)	14 (18)	89 (27)	0.35	0.17-0.71	.004
>12.32	104 (25)	6 (8)	98 (29)	**0**.**14**	**0.05-0.34**	**<**.**001**
Neutrophils (×109/L)						
<4.63	99 (24)	36 (48)	63 (19)	1		
4.63-6.79	104 (26)	27 (36)	77 (23)	0.61	0.34-1.12	.111
6.80-9.95	101 (25)	5 (7)	96 (29)	**0**.**09**	**0.03-0.25**	**<**.**001**
>9.95	104 (26)	7 (9)	97 (29)	**0**.**13**	**0.05-0.30**	**<**.**001**
Lymphocytes (×109/L)						
<0.78	96 (25)	14 (20)	82 (26)	1		
0.78-1.10	97 (25)	9 (13)	88 (28)	0.60	0.25-1.46	.259
1.11-1.60	96 (25)	22 (31)	74 (23)	1.74	0.83-3.65	.142
>1.60	99 (26)	26 (37)	73 (23)	2.09	1.01-4.30	.046
Platelets (×109/L)						
<192	99 (24)	20 (26)	79 (24)	1		
192-254	104 (25)	26 (34)	78 (23)	1.32	0.68-2.55	.415
255-346	106 (26)	21 (28)	85 (25)	0.98	0.49-1.94	.944
>346	102 (25)	9 (12)	93 (28)	0.38	0.17-0.89	.025
Neutrophil/lymphocyte ratio						
<3.43	94 (24)	38 (54)	56 (18)	1		1
3.43-6.13	96 (25)	14 (20)	82 (26)	**0**.**52**	**0.13-0.51**	**<**.**001**
6.14-10.82	99 (26)	15 (21)	84 (27)	**0**.**26**	**0.13-0.52**	**<**.**001**
>10.82	97 (25)	4 (6)	93 (30)	**0**.**06**	**0.02-0.19**	**<**.**001**
Platelet/lymphocyte ratio						
<146.13	95 (25)	26 (38)	69 (22)	1		
146.13-229.20	97 (25)	22 (32)	75 (24)	0.78	0.40-1.50	.454
229.21-357.56	96 (25)	15 (21)	81 (26)	0.49	0.24-1.01	.051
>357.56	98 (25)	6 (9)	92 (29)	0.17	0.07-0.44	<.001
Creatinine ((μmol/L)						
<56	99 (25)	9 (12)	90 (28)	1		
56-72	102 (25)	20 (26)	82 (25)	2.44	1.05-5.66	.038
73-95	100 (25)	16 (21)	84 (26)	1.91	0.80-4.54	.146
>95	102 (25)	33 (42)	69 (21)	**4**.**78**	**2.15-10.65**	**<**.**001**
Sodium (mmol/L)						
<133	83 (21)	8 (11)	76 (23)	1		
133-135	98 (24)	12 (16)	89 (27)	1.31	0.51-3.37	.58
136-138	105 (26)	17 (22)	90 (27)	1.81	0.74-4.43	.193
>138	115 (29)	39 (51)	78 (23)	**4**.**81**	**2.11-10.98**	**<**.**001**
Potassium (mmol/L)						
<4.0	96 (24)	16 (21)	80 (25)	1		
4.0-4.2	83 (21)	20 (26)	63 (19)	1.59	0.76-3.31	.218
4.3-4.6	110 (27)	21 (28)	89 (27)	1.18	0.58-2.42	.651
>4.6	113 (28)	19 (25)	94 (29)	1.01	0.49-2.10	.977

*P*-value from logistic regression.

Bold – selected for multivariable analysis. The level of significance corrected by the Bonferroni method was <0.002.

CI=confidence interval; OR=odds ratio.

In the multivariable analysis, by applying the Variance Inflation Factor for multicollinearity, the number of leucocytes was excluded due to the value >5. In the multivariable analysis, patients with a hemoglobin ≥ 10.6 g/dL and a creatinine level >95 µmol/L had a higher probability of living more than 1 year (Table 4). In contrast, patients with abnormal cognition, pain, anorexia, liver metastases, an ECOG performance status >1, and a neutrophil/lymphocyte ratio ≥ 3.43 had a low probability of living more than 1 year.

**Table 4.** Multivariable Analysis by Logistic Regression.

Variable	OR	95% CI	*P*-value
Cognition			
Normal	1		
Abnormal	**0**.**19**	**0.06-0.65**	.**008**
Pain			
No	1		
Yes	**0**.**42**	**0.20-0.91**	.**027**
Anorexia			
No	1		
Yes	**0**.**35**	**0.15-0.80**	.**013**
Liver metastases			
No	1		
Yes	**0**.**19**	**0.07-0.50**	**<**.**001**
EGOG performance status			
1	1		
2	**0**.**34**	**0.12-0.98**	.**045**
3	**0**.**29**	**0.11-0.81**	.**018**
4	0.32	0.09-1.18	.087
Hemoglobin (g/L)			
<9.1	1		
9.1-10.5	2.71	0.71-10.33	.144
10.6-11.9	**5**.**05**	**1.41-18.05**	.**013**
≥ 12.0	**5**.**45**	**1.53-19.37**	.**009**
Neutrophil/lymphocyte ratio			
<3,43	1		
3.43-6.13	**0**.**17**	**0.07-0.42**	**<**.**001**
6.14-10.82	**0**.**18**	**0.07-0.50**	**<**.**001**
>10.82	**0**.**05**	**0.02-0.19**	**<**.**001**
Creatinine (mmol/L)			
<56	1		
56-72	1.12	0.36-3.50	.842
73-95	0.94	0.30-3.09	.925
>95	**4**.**96**	**1.58-15.56**	.**006**

*P*-value from logistic regression.

Bold—statistically significant.

CI=confidence interval; ECOG=Eastern Cooperative Oncology Group; OR=odds ratio.

## Discussion

Few studies report the number of patients surviving more than 1 year in palliative care, but there are a few examples, such as the study of Good *et al*, which reported 6.1%,^7^ and the Christakis *et al* study, which registered 8.2%.^8^ The percentage observed in this study of 6.4% was similar.

Many variables were associated with survival in the univariable analysis. The remaining elements in the multivariable analysis were cognition, pain, anorexia, liver metastases, ECOG performance status, the number of symptoms, the neutrophil count, the neutrophil/lymphocyte ratio, hemoglobin, and creatinine. Although cancer type could not be included in the multivariable analysis, it seems to be a critical factor, as some are not represented in patients with survival of more than 1 year. Therefore, the probability that a patient with one of those diagnoses survives more than 1 year after suspending antineoplastic treatments is very low.

The prognostic value of symptoms has been extensively studied in patients with advanced cancer with heterogeneous results. The most frequently evaluated symptoms were pain, anorexia, dyspnea, fatigue, nausea, and cachexia/weight loss.^9^ In this study, only pain and anorexia were independent predictors of survival in the multivariable analysis. However, in most studies, pain was not a strong predictor of survival.^9^ The association between symptoms and survival generally seems small (hazard ratios close to 1), except for physical function.^10^

The performance status is a recognized important prognostic factor. The ECOG performance status scale is probably one of the most used in cancer, including advanced cancer.^11–13^ In this study, an ECOG score > 1 was negatively related to survival, as in other studies.^10,11,13^ The cognitive function also had a significant statistical association with survival, as patients with impaired cognition have a lower probability of living more than 1 year. The influence of cognition on prognosis is well known,^9^ and cognitive failure is an independent predictor of survival,^9^ as in this study.

The extension of the disease has a prognostic value. Metastases to the liver, brain, or viscera were associated with shorter survival and cancer bulk, such as the number of metastases, metastatic sites, or tumor volume.^14^ In this study, liver metastases and the number of sites of metastases were associated with survival in the univariable analysis. However, only liver metastases remained statistically significant in the multivariable analysis. In a recent study that included a massive number of patients, liver metastases, even when present at the diagnosis, were associated with a median survival of only 4 months, with significant variations among different primary cancers.^15^ Also, in a study on patients with cancer receiving hospice care in a hospital, liver metastases were an independent factor of poor survival.^16^

A high neutrophil/lymphocyte ratio has a negative prognostic impact on survival, as tens of papers showed. However, there is also heterogeneity among the values considered as cut-offs.^17^ In this study, values of 3.43 and higher were significantly associated with a lower probability of living more than 1 year than lower values. Elevated neutrophil/lymphocyte ratio is a marker of inflammation, indicating a poorer prognosis in patients with cancer. Most cases of cancer-related anemia also result from inflammation,^18^ and anemia is recognized as an adverse prognostic factor in patients with cancer.^19,20^ According to those data, in this study, higher hemoglobin levels were associated with more prolonged survival.

A serum creatinine level >95 µmol/L was significantly associated with survival more prolonged than 1 year relative to lower values. Creatinine is generated in the skeletal muscle at a constant rate.^21^ It depends on the muscle mass. Therefore, a low serum creatinine level reflects a poor nutritional status. Several studies have shown that a low creatinine level is associated with reduced survival. For example, a study showed that a low serum creatinine concentration at admission in hospitalized patients was associated with increased mortality.^22^ In that study, the mortality associated with a very low creatinine value exceeded the mortality of a markedly increased creatinine value. Another study showed that low pre-operative serum creatinine is associated with poor outcomes after nonemergent inpatient surgery.^21^

This study has several limitations related to its retrospective design. Only data recorded in the patients’ files could be retrieved. The data were not systematically collected, namely the symptoms. Also, some biochemical parameters that proved to have prognostic value could not be included in the analysis, such as albumin or reactive C-protein, which are markers of inflammation. Nevertheless, the statistically relevant factors make sense but should be confirmed in a prospective study. Also, the survival criterion for choosing controls may be criticized, but 6 months was considered in the definition of terminal cancer,^6^ although presently, that time frame is not used anymore.^23^ Nevertheless, for this study, it seemed to make sense to maintain that criterion to separate the 2 groups of patients; the intention was to define 2 groups of patients with clear survival differences, excluding possible differences of a few days in the survival of some patients.

## Conclusion

The patients with cancer most likely to survive more than 1 year after admission to palliative care are those with mild or no anemia, a creatinine not low, with an ECOG performance status lower than 2, with normal cognition, without pain or anorexia nor liver metastases, and a low neutrophil/lymphocyte ratio. Cancer type may also influence survival.
